# HLA-DPB1*03 as Risk Allele and HLA-DPB1*04 as Protective Allele for Both Early- and Adult-Onset Multiple Sclerosis in a Hellenic Cohort

**DOI:** 10.3390/brainsci10060374

**Published:** 2020-06-16

**Authors:** Maria Anagnostouli, Artemios Artemiadis, Maria Gontika, Charalampos Skarlis, Nikolaos Markoglou, Serafeim Katsavos, Konstantinos Kilindireas, Ilias Doxiadis, Leonidas Stefanis

**Affiliations:** 1 Faculty of Neurology in Demyelinating Disease Unit & Director of Immunogenetics Laboratory, 1st Department of Neurology, Medical School, National and Kapodistrian University of Athens, NKUA, Aeginition Hospital, 115 28 Athens, Greece; 2 Immunogenetics Laboratory, 1st Department of Neurology, Medical School, National and Kapodistrian University of Athens, NKUA, Aeginition Hospital, 115 28 Athens, Greece; kmwartem@yahoo.com (A.A.); mary212009@windowslive.com (M.G.); charskarlis@med.uoa.gr (C.S.); nmarkoglou@yahoo.gr (N.M.); serafkatsavos@gmail.com (S.K.); 3 Medical School, University of Cyprus, Nicosia 1678, Cyprus; 4 Demyelinating Diseases Unit, 1st Department of Neurology, Medical School, National and Kapodistrian University of Athens, NKUA, Aeginition Hospital, 115 28 Athens, Greece; kildrcost@med.uoa.gr; 5 Transplantation Immunological Laboratory, Institute of Transfusion Medicine, University Hospital Leipzig, 04103 Leipzig, Germany; ilidox@planet.nl; 6 1st Department of Neurology, Medical School, National and Kapodistrian University of Athens, NKUA, Aeginition Hospital, 115 28 Athens, Greece; lstefanis@med.uoa.gr

**Keywords:** Multiple Sclerosis, early-onset, adult-onset, Human Leucocyte Antigens, immunogenetics, clinical phenotype, clinical outcome, therapeutics

## Abstract

**Background**: Human Leucocyte Antigens (HLA) represent the genetic loci most strongly linked to Multiple Sclerosis (MS). Apart from *HLA-DR* and *HLA–DQ*, *HLA-DP* alleles have been previously studied regarding their role in MS pathogenesis, but to a much lesser extent. Our objective was to investigate the risk/resistance influence of *HLA-DPB1* alleles in Hellenic patients with early- and adult-onset MS (EOMS/AOMS), and possible associations with the HLA-DRB1*15:01 risk allele. **Methods**: One hundred MS-patients (28 EOMS, 72 AOMS) fulfilling the McDonald-2010 criteria were enrolled. HLA genotyping was performed with standard low-resolution Sequence-Specific Oligonucleotide techniques. Demographics, clinical and laboratory data were statistically processed using well-defined parametric and nonparametric methods and the SPSSv22.0 software. **Results**: No significant *HLA-DPB1* differences were found between EOMS and AOMS patients for 23 distinct *HLA-DPB1* and 12 *HLA-DRB1* alleles. The HLA-DPB1*03 allele frequency was found to be significantly increased, and the HLA-DPB1*02 allele frequency significantly decreased, in AOMS patients compared to controls. The HLA-DPB1*04 allele was to be found significantly decreased in AOMS and EOMS patients compared to controls. **Conclusions**: Our study supports the previously reported risk susceptibility role of the HLA-DPB1*03 allele in AOMS among Caucasians. Additionally, we report for the first time a protective role of the HLA-DPB1*04 allele among Hellenic patients with both EOMS and AOMS.

## 1. Introduction

Multiple sclerosis (MS) is considered a complex, multifactorial disease entity, as both environmental and genetic factors have been implicated in its pathogenesis [1]. The Major Histocompatibility Complex (MHC) represents a cluster of highly polymorphic genes, including mainly the Human Leukocyte Antigens (HLA) system, namely Class I (A, B, C) and II (DR, DQ, DP) genes, and genes encoding for some other immune factors, like complement components, Bf, C2, C4 and TNF, in Class III and IV loci [2]. HLA molecules mediate antigen presentation to T-lymphocytes, playing a crucial role in immune response and affecting all clinical and neuroimaging characteristics and response to treatment in MS [3,4]. Linkage studies in various populations have consistently demonstrated that the MHC and its polymorphisms represent the genetic locus most strongly linked to MS [3,4,5], and that the MHC class II (*HLA-DR, HLA-DQ, HLA-DP*) region is the susceptibility complex that accounts for the majority of familial clustering in MS [6].

The MHC class II linkage to MS differs in various populations, with the highest association conferred by the HLA-DRB1*15:01/HLA-DQB1*06:02 haplotype, present in Caucasians [5]. In 2011, in a collaborative European study, the HLA-DRB1*15:01 allele exhibited the strongest association with MS, along with the HLA-DRB1* 03:01 and HLA-DRB1*13:01 alleles [7], although DRB1*15:01 was recently found to be hypomethylated and predominantly expressed in monocytes among carriers of DRB1*15:01, suggesting putative therapeutic strategies targeting methylation-mediated regulation of this major risk gene [8].

Recent studies have further established the role of HLA-DRB1*15:01 in early-onset (pediatric and adolescent) MS (EOMS), which accounts for 3–5% of all MS cases, while the role of HLA-DRB1*04 and HLA-DRB1*03 remains to be clarified [9,10,11].

Apart from the well examined *HLA-DR* and *HLA-DQ* genes, other class II genes and their products, *HLA-DP* alleles, have been previously studied regarding their role in MS pathogenesis. One of the earliest studies regarding *HLA-DP* genotyping was performed three decades ago using a small sample of 45 Swedish patients with MS in comparison with 166 Danish controls [12]. Since then, few studies have been published on the role of the *HLA- DPB1* locus concerning genetic risk in adult-onset MS (AOMS), either in Asian [13,14,15,16] or European populations [17,18,19,20,21], and no such studies have been performed on EOMS. In 2013, Patsopoulos et al. used single nucleotide polymorphisms (SNP) data from genome-wide studies and tested classical alleles and polymorphisms in eight classical HLA genes in 5091 AOMS cases and 9595 controls [22]. Among a total of 11 identified statistically independent effects, they confirmed a possible association of HLA-DPB1*03:01, and also highlighted a more statistically significant effect at amino acid position 65 in the peptide binding groove of HLA-DPB1* [22]. So far, HLA-DPB1* alleles have been mainly correlated with neuromyelitis optica spectrum disorders (NMOSD) in Asian but not Caucasian populations [23], while a series of studies suggest a possible role in other autoimmune disorders as well, including juvenile idiopathic arthritis [24], type I diabetes [25] and atopic myelitis in Japanese [26].

The present study attempts to expand the existing data on HLA and MS by investigating the influence of HLA-DPB1* alleles on disease risk and resistance in a Hellenic sample of 100 patients of both EOMS and AOMS, using healthy controls (HC) for comparisons, given the pre-existing difference in *HLA-DRB1* allele frequencies in EOMS and AOMS in our ethnic group [11] and the total absence of information on *HLA-DPB1* genotyping in the Hellenic MS population.

Additionally, we examined, the putative positive or negative association between the well-defined HLA-DRB1*15:01 allele and the various HLA-DPB1* alleles, given the extensive epistatic mechanisms that exist in HLA loci, as clearly illustrated in previous reports [12,19,27,28].

## 2. Materials and Methods

### 2.1. Patients

One hundred patients with MS (62 females, 38 males, mean age 36.9 ± 11.4 years old) were selected, fulfilling the McDonald criteria for MS diagnosis [29]. These patients were enrolled from the outpatient clinic at the Neurology Department of the Aeginition University Hospital (Athens, Greece) after providing written informed consent. The study received ethical approval by the Hospital’s Ethics Committee (ethic approval code number: 117/2-4-13), as it was found consistent with the Declaration of Helsinki. At the time this study, 42 patients had the relapsing-remitting type of the disease (RRMS), and 10 patients were identified with primary progressive MS (PPMS), while the rest had the secondary progressive type (SPMS). For all patients, the mean age of disease onset was 27.8 ± 10.8 years old, the mean disease duration was 100.9 ± 80.4 months and the median Expanded Disability Status Scale (EDSS) was 3.0 (range:1.0–8.0) [30]. There were two MS onset groups; 28 in the ≤19 years old or early onset MS (EOMS) group and 72 in the >19 years old or adult onset MS (AOMS) group. Valid Magnetic Resonance Imaging (MRI) and cerebrospinal fluid (CSF) (i.e., presence of oligoclonal bands and IgG index calculation) assessments were available 60 (60%) of the patients. Missing data for MRIs were attributed to the lack of recent MRI scans. With regards to CSF, some but not all patients had been subjected to CSF analysis, since this was not a prerequisite for the MS diagnosis, according to the revised 2010 McDonald criteria [29]. All patients provided informed consent for participation and publication.

### 2.2. HLA-DPB1* and HLA-DRB1* Genotyping

HLA genotyping was performed at the Immunogenetics Laboratory of the 1st Department of Neurology, in Aeginition Hospital. High molecular weight DNA was extracted from peripheral blood samples (8 mL peripheral blood in sodium citrate, ACD Vacutainer^®^ tube) using the DNA extraction, Maxi Kit (QIAGEN, Venlo, the Netherlands) as per manufacturer’s guidelines in the commercial kit. HLA class II (*HLA-DRB1* and *HLA-DPB1*) frequencies were determined by molecular techniques for all the specificities included in the HLA Nomenclature of 2012 (we present only the first two or four digits of each allele, for low or high resolution respectively) [31]. *HLA-DRB1* genotyping had been previously performed, using a PCR-SSO (Polymerase-Chain-Reaction, PCR, Sequence-Specific Oligonucleotide, SSO) technique (Elpha Bio-Rad, High resolution), as described elsewhere [11]. *HLA-DPB1* genotyping was performed using a different PCR-SSO technique, based on a method that depends on reverse hybridization (Line Probe Assay, INNO-LiPA, Low Resolution, Innogenetics, Fujirebio, Europe) according to the manufacturer’s protocol.

### 2.3. Statistical Analyses

The Hardy-Weinberg proportions (HWP) and linkage disequilibrium for *HLA-DPB1*, *HLA-DRB1* haplotypes were ascertained using the PyPoP software [32]. An Ewens-Watterson (EW) homozygosity test for neutrality was also performed. Calculation of the normalized deviate of the homozygosity (i.e., Fnd) was done, with positive and negative values implying directional and balancing selection, respectively. HLA-DPB1* genotype frequency in patients with MS was compared with that reported in a previous study of Hellenic HC by using multiple binomial tests [33].

Separate analyses were performed in the EOMS and AOMS groups using the same expected genotype frequencies of the healthy controls [33]. A Fisher’s exact test for categorical and Mann-Whitney U test for numerical variables were performed to allow us to make group comparisons. Mantel-Haenszel statistics were used to ascertain the role of MS groups in the association between *HLA-DPB1* genotypes and categorical clinical parameters. In *HLA-DPB1* genotype-related tests (except those for clinical parameters), *p* value correction was made according to the Benjamini–Yekutieli method (or B–Y) based on the following formula: *p* (B–Y) = a/(Σ1/i), where i denotes the number of comparisons and a = 0.05 [34,35]. Statistical analyses were performed using the SPSS v22.0 software (Armonk, NY, USA: IBM Corp).

## 3. Results

### 3.1. HWP and Linkage Disequilibrium of the Study’s Sample

Twenty-three distinct *HLA-DPB1* alleles were identified (total alleles: 200). There were 28 homozygote and 72 heterozygote patients with MS. There were no deviations from the HWP (homozygotes: 29.71 expected, F(1) = 0.1, *p* = 0.754, heterozygotes: 70.29 expected, F(1) = 0.04, *p* = 0.838). The most common haplotypes were HLA-DPB1*04/DPB1*04 (27%), followed by HLA-DPB1*02/DPB1*04 (13%), HLA-DPB1*03/DPB1*04 (11%) and HLA-DPB1*10/DPB1*04 (6%). The EW homozygosity test of neutrality was found to be significantly positive (i.e., Fnd = 3.79, *p* = 0.992, i.e., over the limit 0.975), denoting a directional selection of the HLA-DPB1*04 allele.

Twelve distinct *HLA-DRB1* alleles were identified (total alleles: 174) in 87 out of the 100 patients. There were 11 (12.6%) homozygote and 76 (87.3%) heterozygote patients. There were no deviations from the HWP (homozygotes: 10.84 expected, F(1) = 0, *p* = 0.962, heterozygotes: 76.16 expected, F(1) = 0, *p* = 0.986). The most common allele was HLA-DRB1*11 (20.1%), followed by HLA-DRB1*16 (15.5%), HLA-DRB1*15 (13.2%), HLA-DRB1*04 (12.1%) and HLA-DRB1*13 (10.4%). The most common, but still of low frequency (4.6%), genotype was HLA-DRB1*11/DRB1*16. The EW homozygosity test of neutrality was found to be significantly negative (i.e., Fnd = −1.41, *p* = 0.0033, i.e., lower the limit 0.05), indicating a balancing selection.

The delta distance for the *HLA-DPB1* and *HLA-DRB1* haplotypes was 0.00938 (*p* = 0.303), denoting linkage equilibrium. This did not change when age of MS onset was taken into account (EOMS: delta 0.0128, *p* = 0.954, AOMS: delta 0.0133, *p* = 0.351). The most common (i.e., over 5%) *HLA-DPB1/HLA-DRB1* haplotypes were HLA-DPB1*04/HLA-DRB1*11 (10.8%), HLA-DPB1*04/HLA-DRB1*16 (7.7%), HLA-DPB1*04/HLA-DRB1*04 (7%), HLA-DPB1*02/HLA-DRB1*11 (6.6%) and HLA-DPB1*04/HLA-DRB1*03 (5.5%).

### 3.2. Nongenetic Comparisons between Age of Onset Groups

Table 1 presents the main characteristics of the two MS groups. Patients with EOMS were significantly younger and had longer disease duration compared to AOMS, which primarily reflects the blood sampling timing, and has no specific clinical significance. Of most importance, patients with EOMS had significantly higher IgG indexes compared to AOMS. It should be noted that this difference reflects 60 out of the 100 patients with MS of this study, since, as mentioned in the methods section, no CSF testing was available for 40 patients.

### 3.3. HLA-DPB1 Allele Comparisons between the Age of Onset Groups

No significant *HLA-DPB1* allele differences were found between patients with EOMS and AOMS (Table 2). However, there were significantly fewer HLA-DPB1*04-positive patients in the EOMS group compared to HC (64.3% vs. 92.7%). The HLA-DPB1*03 allele was found to be significantly increased in patients with AOMS compared to HC (23.6% vs. 13.4%). On the other hand, HLA-DPB1*02 and HLA-DPB1*04 were found to be significantly decreased (*p* < 0.001) in patients with AOMS compared to HC (22.2% vs. 36.6% and 79.2% vs.92.7%).

A total of 21 out of 87 patients (24.1%) were positive for the HLA-DRB1*15 allele, which is significantly higher than the expected 11.4% allele frequency in HC (*p* < 0.001), confirming the well-established role of this allele in MS pathogenesis [33]. HLA-DRB1*15 allele positivity was 20.8% (5/24) for EOMS and 25.4% (16/63) for AOMS (*p* = 0.783).

Table 3 presents the HLA-DRB1*15 allele frequency among the different HLA-DPB1* alleles. Only statistically significant associations are presented. The HLA-DRB1*15 allele was statistically significantly absent among HLA-DPB1*03 positive patients (*p* = 0.001) and among HLA-DPB1*03 positive AOMS (*p* = 0.003), whereas it was significantly increased among HLA-DPB1*04 (*p* = 0.048), HLA-DPB1*14 (*p* = 0.008) -positive genotype patients. Finally, the HLA-DRB1*15 allele was positive in the two HLA-DPB1*14 positive patients with EOMS (*p* = 0.036).

**Figure 1:**
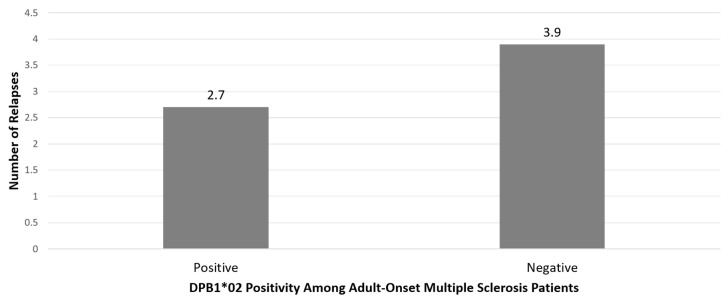
Number of relapses in adult-onset Multiple Sclerosis Patients with regards to HLA-DPB1*02 genotype. Positive patients had significantly fewer relapses than negative (*p* = 0.033).

In the 60 patients with available CSF examination, those with the HLA-DPB1*02 allele had significantly higher IgG indexes than those who were negative for HLA-DPB1*02 (mean 1.22 ± 0.70 vs. 0.75 ± 0.39, respectively, *p* = 0.02), irrespective of age of MS onset. There were no other significant associations between the *HLA-DPB1* or *HLA-DRB1* alleles (i.e., presence or not of each HLA-DPB1* allele and HLA-DRB1*15 allele) and gender, type of MS, MRI or CSF assessments (data not shown). Patients with AOMS who were positive for HLA-DPB1*02 had significantly fewer relapses since onset than HLA-DPB1*02 negative patients with AOMS (2.7 ± 2.5 vs. 3.9 ± 2.4, *p* = 0.033), corroborating the protective role of HLA-DPB1*02 phenotype, as reported above (Figure 1). No other MS group effects on the HLA and clinical parameter associations were found.

## 4. Discussion

HLA-immunogenetics is an old but still rapidly expanding field in MS pathogenesis. In order to keep abreast of rapid developments in this field, we investigated the role of the *HLA-DP* locus in MS pathophysiology. We genotyped 100 Hellenic patients with MS for *HLA-DR* and *HLA-DP* alleles, as described above, which is a rather small sample and the main limitation of this study. *HLA-DPB1* genotyping was performed for the first time on a Hellenic MS population and in patients with EOMS, which is the core novelty of our research, albeit on a small sample (28 patients); however, we highlight again that EOMS is a rare disease entity and represents only the 3–5% of all MS patients in Caucasian populations.

In our study, we replicated the well-established predominance of the HLA-DRB1*15 genotype in Hellenic patients with MS compared to HC, independently of age at disease onset [11].

No statistically significant *HLA-DPB1* allele differences were found between patients with EOMS and AOMS. All statistically significant differences were investigated in the AOMS group, except for the HLA-DPB1*04 allele, which is lower in EOMS and AOMS, compared to the HC group at a high statistical level (*p* < 0.001, Table 2), suggesting a possible protective role in the Hellenic population. This is in contrast with an early study in 1988 [12] where the frequencies of DPw4 were 93.3% in patients with MS and 72.3% in controls (relative risk, *R^2^* = 5.4, *p* = 0.0014). Nevertheless, we have to mention that in this early study, the HLA-DNA typing was carried out on a small sample of 45 patients with MS and 63 controls of different ethnic European groups (Swedish and Danish), using the Restriction Fragment Length Polymorphism (RFLP) technique for *HLA-DP* and *HLA-DR* genes. In this same study, the *HLA-DR2* antigen was present in 75.5% of patients and in 33.7% of the controls (*R*^2^ = 6.1, *p* less than 10(-6)). *HLA-DPw4* was not associated (i.e., was not in linkage disequilibrium) with *HLA-DR2* in patients or controls. Thus, the researchers concluded that in MS, the associations with *HLA-DP* and *HLA-DR* are independent of each other, but the combined presence of *HLA-DPw4* (cellularly defined) and *HLA-DR2* represented a significantly higher risk than either antigen alone, indicating that synergism between *HLA-DP* and *HLA-DR* gene products may play a role in the genetic susceptibility to MS. On the other hand, a recent study on celiac disease showed that the HLA-DPB1*04:01 allele protects genetically-susceptible children from celiac disease [36], a fact that is in line with our results, concerning children and adults with MS, while in another study in 2015, another HLA-DPB1*04 allele, namely HLA-DPB1*04:02, conferred a strong protective effect against narcolepsy [37]. Finally, the worldwide risk HLA-DRB1*15 allele in MS, in Caucasians, was found to be significantly increased among HLA- DPB1*04 positive patients with MS (*p* = 0.048) in our sample.

In another early study in France in 1991, it was found that the distribution of *HLA-DPB1* alleles was not significantly different in patients with MS and controls [20]. Nowadays, it is perfectly clear that the HLA-DP*03 allele is associated with MS and epitope spreading in MS [17,22], and in this study, we observed the risk susceptibility of this allele in our Hellenic MS sample, at a highly significant level (*p* < 0.009, Table 2).

Regarding AOMS, the HLA-DPB1*03 allele could be a risk factor for the disease, as it was found to be significantly increased in patients with AOMS compared to HC. The percentage of HLA-DPB1*03 positive patients with EOMS was higher than HC (17.9% vs. 13.4%), although at a nonstatistically significant level. The HLA-DRB1*15 allele was absent among HLA-DPB1*03 positive patients. This cannot be attributed to linkage disequilibrium, as this was tested. Linkage disequilibrium for the *HLA-DR* and *HLA-DP* genes was excluded in previous studies as well [12]. Since HLA-DPB1*03 was found to be increased in AOMS, it may constitute a risk factor; this genotype may exert its risk factor effect only in the absence of HLA-DRB1*15, at least in AOMS. Moreover, the HLA-DRB1*15 allele was found to be significantly increased among HLA-DPB1*04-positive patients, suggesting that HLA-DPB1*04 exerts a protective effect only in the absence of HLA-DRB1*15. Despite the relatively small sample size in our study, these findings suggest that epistatic mechanisms between Class II *HLA-DR* and *HLA-DP* alleles may play a role in disease pathogenesis and risk of disease occurrence. This conclusion is in line with the results of Dekker et al. [19] who observed that in patients with MS who lacked HLA-DQB1*06:02 allele, the HLA-DPB1*03:01 allele frequency was significantly (*p* = 0.006) increased (50.0%) compared with HLA-DQB1*06:02-negative controls (9.1%). In parallel, in 2009, Lincoln et al. highlighted the role of epistasis between HLA-DRB1*15 and HLA-DQA1*01:02 alleles. More specifically, they proved that HLA-DQA1*01:02, which shows no primary MS association, increases disease risk when combined with HLA-DRB1*15:01, through transepistatic interactions [27]. Of note is the fact that the presented slight *HLA-DPB1* allele differences between AOMS and EOMS could also reflect the different clinical course of these two groups, given that patients with older age at onset are known to be more at risk of having secondary-progressive disease. For instance, predicting the onset of secondary-progressive multiple sclerosis is accomplished using genetic and nongenetic factors, with the HLA-A*02:01 allele conferring a decreased risk for MS and also contributing to decreased hazards for SPMS [38].

Another finding in our study that is worthy of mention is the possible protective role of the HLA-DPB1*02 allele in AOMS. HLA-DPB1*02 was found to be significantly decreased in AOMS, while those who were HLA-DPB1*02 positive had, in general, fewer relapses since onset compared to HLA-DPB1*02-negative patients with AOMS, corroborating the protective role of the HLA-DPB1*02 allele reported above (Figure 1).

The HLA-DPB1*35 allele was found to be significantly increased in patients with AOMS compared to HC, while increased prevalence of the HLA- DRB1*15 allele in HLA-DPB1*14-positive patients with MS and patients with EOMS was also noted. Nevertheless, their possible genetic risk should be interpreted with caution, due to the very low frequency of these alleles.

At this point, we have to mention that the HLA-DPB1*04 allele is the most frequent in the Hellenic population (92.7%), followed by HLA-DP*02 (36.6%) and HLA-DP*03 (13.4%) [30]. Additionally, according to our results, the most common *HLA-DPB1-*haplotypes in Hellenic patients with MS were HLA-DPB1*04/DPB1*04 (27%), followed by HLA-DPB1*02/DPB1*04 (13%), HLA-DPB1*03/DPB1*04 (11%) and HLA-DPB1*10/DPB1*04 (6%). Thus, the emerging protective role of the HLA-DP*04 allele is in parallel with the HLA-DR*11 allele, which is the most common in the Hellenic population, and the protective *HLA-DRB1* allele in Hellenic patients with MS [11].

The role of *HLA-DPB1* alleles has been studied in a range of other autoimmune diseases, especially NMOSD [23]. More specifically, HLA-DPB1*05:01, which is extremely rare in Caucasian populations, has the strongest association with opticospinal MS and anti-AQP4 seropositivity in Asian populations, while HLA-DPB1*03 possibly offers genetic protection against the disease [23]. Moreover, HLA- DPB1*02:01 has been associated with oligoarticular and rheumatoid factor-negative polyarticular juvenile idiopathic arthritis and childhood-onset diabetes type I in the Japanese population [24,25].

Therapeutic interventions in MS are sometimes difficult, because the patient’s symptoms at the initial stages are not clearly suggestive of a definite demyelinating syndrome, especially in children. Furthermore, sometimes the neuroradiological (MRI) aspects and blood antibody tests are not helpful. In these situations, having a marker or a combination of markers that supports the differential diagnosis is of crucial importance, and has a direct impact on therapeutic decision making. The *HLA-DR* alleles, and especially the HLA-DR*15 allele, are the most robust genetic markers for almost every clinical or paraclinical aspect of the disease [4] in Caucasians and for the therapeutic response to different Disease Modified Treatments (DMTs) [4]. Nowadays, the expansion and overlap of various demyelinating diseases, namely MS, NMOSD, ADEM (Acute Disseminating Encephalomyelitis), MOG-Demyelinating (Myelin Oligodendrocyte Glycoprotein-Demyelinating) disease, Optic Neuritis, etc., make the need for specific biomarkers more urgent than ever before, as noted in our previous critical review [39] and in this works of other researchers [40].

Apart from a genetic association with MS and other demyelinating diseases, *HLA-DP* molecules play a key role in MS pathogenesis and progression, as described many years ago [17,41].

Additionally, in our Hellenic cohort, the *HLA-DP* alleles seemed to play an independent role in patients with MS (risk/protective), apart from the *HLA-DR* alleles, a fact that has to be confirmed in larger cohorts in the future. This could pave the way for the usage of these alleles in patient stratification (carriers and noncarriers)—as already happens with various HLA-DRB1 alleles and especially with the HLA-DRB1*15 allele [4,42]—for many MS characteristics and therapy responses in different DMTs in Caucasian populations [4,42].

Altogether, clarification of *HLA-DP* allele associations with both EOMS and AOMS is needed in every ethnic group to get a better idea of clinical features and MS phenotypes and disease progression, and as a form of future putative data for better therapeutics.

## 5. Conclusions-Limitations

In conclusion, our study supports the previously reported risk susceptibility role of the HLA-DPB1*03 allele in AOMS in many Caucasian populations. Additionally, we report, for the first time in the international literature, the protective role of the HLA-DPB1*04 allele for patients with both EOMS and AOMS, and the putative protective role of the HLA-DPB1*02 allele in patients with AOMS in our sample. Another finding that is worthy of mention is the total absence of the well-established HLA-DRB1*15 allele among patients having the most statistically frequent HLA-DPB1*03 allele in our cohort.

A limitation of our study was the relatively small sample size (28 patients with EOMS and 72 patients with AOMS). Indeed, observed small effect sizes for DPB1 alleles from the different group comparisons were the following: EOMS vs. AOMS 7%, EOMS vs. HCs 21.9% and AOMS vs. HCs 25.8%. However, this is a first attempt towards clarifying the role of the *HLA-DPB1* alleles in MS in a Hellenic AOMS and EOMS cohort. Moreover, the small study sample did not allow us to conduct multivariable analyses, which would more readily reveal confounding effects in our analyses.

These novel data could also contribute to personalized MS-therapeutics in the near future, taking into account the rapid expansion of our knowledge of multiple sclerosis and other distinct demyelinating diseases in many ethnic groups.

## Figures and Tables

**Table 1:** Nongenetic Comparisons Between Age of Multiple Sclerosis (MS) Onset Groups.

Characteristics	EOMS	AOMS	Sig ^1^
Females	19/28 (67.9%)	43/72 (59.7%)	0.499
Age (years old)	29.9 ± 9.8	39.8 ± 10.8	0.001 *
Duration of MS (months)	148.1 ± 105.4	81.3 ± 69.1	0.011 *
EDSS	3.1 ± 1.7	3.3 ± 1.6	0.414
Primary Progressive	3/28 (10.7%)	7/72 (9.7%)	0.917
Relapses since onset	5.2 ± 6.1	3.7 ± 2.5	0.393
IgG index ^2^	1.3 ± 0.7	0.8 ± 0.4	0.004 *
Presence of OCBs ^2^	13/15 (86.7%)	31/45 (68.9%)	0.312
Subcortical lesions ^2^	12/17 (70.6%)	27/43 (62.8%)	0.765
Periventricular lesions ^2^	16/17 (94.1%)	42/43 (97.7%)	0.49
Infratentorial lesions ^2^	12/17 (70.6%)	30/43 (69.8%)	1.000
Spinal cord lesions ^2^	12/14 (85.7%)	29/31 (93.5%)	0.578

Numbers represent means ± standard deviation and absolute (%) frequencies; 1 Fisher exact test for categorical and Mann-Whitney U test for numerical characteristics. OCBs: Oligoclonal Bands, Sig.: significance. 2 Valid MRI and cerebrospinal fluid assessments were available for 60 (66%) and 60 (60%) patients, respectively. * *p* ≤ 0.05.

**Table 2:** *HLA-DPB1* Allele Frequencies Among Different Age of Multiple Sclerosis (MS) Onset Groups of Patients.

Early MS		Adult MS	HCs	Early vs. Adult MS	Early MS vs. HCs	Adult MS vs. HCs
	(N = 28)	(N = 72)	(N = 246)	Sig ^1^	Sig ^2^	Sig ^2^
HLA-DPB1*01	3.6	4.2	4.5	0.85 (0.09–8.55) 1.000	0.79 (0.1–6.37) 0.500	0.92 (0.25–3.42) 0.500
HLA-DPB1*02	39.3	22.2	36.6	2.27 (0.89–5.80) 0.131	1.12 (0.5–2.5) 0.461	0.5 (0.23–0.91) 0.008 **
HLA-DPB1*03	17.9	23.6	13.4	0.7 (0.23–2.13) 0.602	1.4 (0.50–3.95) 0.339	2.0 (1.04–3.84) 0.009 **
HLA-DPB1*04	64.3	79.2	92.7	0.47 (0.18–1.24) 0.132	0.14 (0.06–0.35) <0.001 **	0.3 (0.14–0.63) <0.001 **
HLA-DPB1*05	3.6	2.8	2.4	1.3 (0.11–14.89) 1.000	1.48 (0.17–12.77) 0.500	1.14 (0.23–5.79) 0.500
HLA-DPB1*06	0	1.4	0.8	- 1.000	- 0.500	1.72 (0.15–19.23) 0.500
HLA-DPB1*09	0	1.4	2.8	- 1.000	- 0.372	0.48 (0.06-3.97) 0.356
HLA-DPB1*10	10.7	9.7	4.9	1.11 (0.27–4.65) 1.000	2.34 (0.62–8.85) 0.162	2.1 (0.8–5.55) 0.052
HLA-DPB1*11	0	1.4	-	- 1.000	-	-
HLA-DPB1*13	3.6	2.8	6.1	1.3 (0.11–14.89) 1.000	0.57 (0.07–4.49) 0.435	0.44 (0.1–1.97) 0.176
HLA-DPB1*14	7.1	6.9	3.7	1 (0.18–5.48) 1.000	2.03 (0.42–9.88) 0.321	1.97 (0.64–6.06) 0.126
HLA-DPB1*15	0	4.2	2.0	- 0.557	- 0.468	2.1 (0.49–8.99) 0.186
HLA-DPB1*19	0	1.4	0.4	- 1.000	- 0.500	3.45 (0.21–55.87) 0.346
HLA-DPB1*22	3.6	0	-	- 0.280	-	-
HLA-DPB1*23	3.6	0	2.4	- 0.280	1.48 (0.17–12.77) 0.500	- 0.172
HLA-DPB1*32	3.6	0	0.8	- 0.280	4.52 (0.4–51.49) 0.279	- 0.460
HLA-DPB1*33	3.6	2.8	0.4	1.3 (0.11–14.89) 1.000	9.07 (0.55–149.24) 0.123	7.0 (0.63–78.34) 0.130
HLA-DPB1*34	0	1.4	-	- 1.000	-	-
HLA-DPB1*35	0	4.2	0.8	- 0.557	- 0.500	10.65 (1.09–104.03) 0.005 **
HLA-DPB1*38	3.6	0	-	- 0.280	-	-
HLA-DPB1*46	3.6	0	-	- 0.280	-	-
HLA-DPB1*50	0	1.4	0.4	- 1.000	- 0.500	3.45 (0.21–55.87) 0.346
HLA-DPB1*56	0	1.4	-	- 1.000	-	-

Numbers represent frequencies (%). 1 Fishers’s exact test (23 comparisons) 2 Binomial tests (17 comparisons). ** *p* ≤ 0.015 (17 comparisons) or *p* ≤ 0.013 (23 comparisons), according to the Benjamini–Yekutieli method for 17 comparisons, HCs: Healthy Control

**Table 3:** Significant DRB1*15 Positivity Differences among *HLA-DPB1* Alleles and Age of Onset Groups in Multiple Sclerosis (MS) Patients.

**Total Sample of MS Patients**		
HLA-DPB1* Genotype	HLA-DRB1*15 Positive	Sig ^1^
HLA-DPB1*03	0/22 (0%)	0.001 *
HLA-DPB1*04	19/63 (30.2%)	0.048 *
HLA-DPB1*14	5/7 (71.4%)	0.008 *
**Adult-Onset MS**		
HLA-DPB1* Genotype	HLA-DRB1*15 Positive	Sig ^1^
HLA-DPB1*03	0/17 (0%)	0.003 *
**Early-Onset MS**		
HLA-DPB1* Genotype	HLA-DRB1*15 Positive	Sig ^1^
HLA-DPB1*14	2/2 (100%)	0.036 *

Values represent observed frequencies (%) of HLA-DRB1*15 allele positivity among the different *HLA-DPB1* genotypes. 1 Fisher exact tests. * *p* ≤ 0.05.

## Data Availability

The datasets generated during and/or analyzed during the current study are available from the corresponding author on request.
